# You say it‘s not me: the influence of offering external explanations of rejection and acceptance behavior on the perception of benevolence in borderline personality disorder

**DOI:** 10.1186/s40479-024-00275-y

**Published:** 2024-11-25

**Authors:** Anna Schulze, Berit Rommelfanger, Elisabeth Schendel, Kornelius Immanuel Kammler-Sücker, Stefanie Lis

**Affiliations:** 1 Department of Clinical Psychology, Central Institute of Mental Health, Medical Faculty Mannheim, University of Heidelberg, Mannheim, Germany; 2 Center for Innovative Psychiatric and Psychotherapeutic Research (CIPP), Central Institute of Mental Health, Medical Faculty Mannheim, University of Heidelberg, Mannheim, Germany; 3 Institute of Cognitive and Clinical Neuroscience, Central Institute of Mental Health, Medical Faculty Mannheim, University of Heidelberg, Mannheim, Germany; 4 Department of Psychosomatic Medicine and Psychotherapy, Central Institute of Mental Health, Medical Faculty Mannheim, University of Heidelberg, Mannheim, Germany

**Keywords:** Borderline Personality Disorder, Rejection, Acceptance, Attribution, Virtual reality

## Abstract

**Background:**

Interpersonal impairments in patients diagnosed with borderline personality disorder (BPD) are characterized by the fear of being rejected and high levels of loneliness. Potential underlying factors are alterations in the processing of social interactions and the associated perceptions of social partners. In this regard, BPD patients tend to attribute the cause of negative rather than positive events to their own person and to perceive others as less trustworthy than healthy controls (HCs). To date, no study has investigated whether the effect of experimentally influenced causal attributions of social interactions on the perception of a social partner differs between BPD patients and HCs.

**Methods:**

A new virtual reality paradigm was developed to investigate the perception of benevolence following the induction of social rejection and acceptance, while experimentally manipulating whether an external cause for this behavior was provided. The data of 62 participants (32 HCs, 30 BPD patients) were analyzed using linear mixed-effects models. Associations of benevolence ratings with attributional style, rejection sensitivity, self-esteem, childhood trauma, and loneliness were investigated via correlational and multiple linear regression analyses.

**Results:**

Across both groups, a social partner was rated as less benevolent following rejection than following acceptance. An external explanation mitigated this negative effect of rejection. Overall, benevolence ratings were lower in BPD patients than in HCs. This group difference was stronger following acceptance than following rejection. Independent of acceptance and rejection, an external explanation was associated with a higher level of benevolence only in the HC group. No associations of the effects of the experimental conditions with attributional style, childhood trauma, rejection sensitivity, self-esteem, or loneliness were found.

**Conclusion:**

Our findings indicate that acceptance and provided external explanations for rejection have a less positive impact on the perception of a social partner’s attitude toward oneself in BPD patients than in HCs. More research is needed to identify predictors of benevolence perception and which steps of social information processing are altered. The therapeutic implications include the importance of strengthening the perception and enjoyment of being accepted as well as improving the mentalizing ability of BPD patients.

**Supplementary Information:**

The online version contains supplementary material available at 10.1186/s40479-024-00275-y.

## Background

 Personality functioning in borderline personality disorder (BPD) is characterized by impairments in interpersonal functioning, such as anxious preoccupation with real or imagined abandonment and mistrust toward the intentions of others, often combined with impairments in self-functioning including excessive self-criticism and a low self-esteem [1]. These problems can be observed in empirical studies showing high levels of loneliness and distrust toward others, a greater tendency to feel socially excluded and rejected, and alterations in self-referential processing including a maladaptive attributional style [2–5]. Although it seems theoretically obvious that these changes in self and interpersonal functioning are closely related, empirical studies on the interplay of these processes are sparse. The current study aimed to contribute to the understanding of the interplay between inferring social causality and social judgments in interpersonal encounters in BPD patients. Using a virtual reality approach, we investigated the effects of social rejection and acceptance on the evaluation of the trustworthiness of others and whether these social judgments can be influenced by providing an explanation for the interaction partner’s behavior.

### Social rejection and BPD

Being rejected by others violates the need to belong to one of the basic human needs [6]. Experiencing social rejection has been linked to multifaceted changes in both intra- and interindividual processes that might result in a heightened motivation to strengthen social relationships, emotional numbness or even aggressive behavior, with differential consequences for achieving the desired social closeness [7, 8].

The experience of social rejection has been one focus of research on social cognition in BPD patients (for review see [9]). Studies have consistently found particularly high levels of rejection sensitivity, that is, a personality disposition to anxiously expect social rejection [10], in BPD patients even compared with clinical control groups (e.g. [11, 12]). A stronger experience of social exclusion has also been shown in experimental studies. Studies using script-driven imagery paradigms to induce rejection revealed an association between BPD and enhanced emotional reactivity toward rejection [13, 14]. Studies contrasting the experience of social exclusion and acceptance have mostly used the cyberball paradigm [15]. In this virtual ball-tossing game, the proportion of ball tosses the participant receives while exchanging ball throws with co-players induces social exclusion and inclusion. In BPD, higher levels of experienced social exclusion were found across most experimental conditions including even conditions with an overinclusion of the participants or ball-tossing sequences that were determined by predefined rules instead of by the intentions of the alleged co-players [12, 16, 17]. These findings indicate that not only differences in perceptions of rejection but also of acceptance are important for understanding interpersonal difficulties in BPD patients.

Experimental studies using the cyberball paradigm have focused on the processing of ostracism that is being socially excluded or ignored. Although social exclusion is a painful experience and is associated with various detrimental short- and long-term consequences (for review see [18]), it is different from the concept of social rejection. Accordingly, Peng et al. [19] showed that cognitive control processing is influenced differently by social rejection than by exclusion. In the literature, social rejection often refers to being explicitly not wanted after joint interaction and is conceptualized as a form of relational evaluation [20]. Although this approach resembles the definition of rejection sensitivity as operationalized in the commonly used rejection sensitivity questionnaire to a greater extent [21], this definition does not specify the inner dynamic or behavior of the rejected person in the joint interaction. The focus of the questionnaire is on social situations in which an individual reveals themselves to be in need of or wanting something. This conceptualization of rejection therefore goes beyond the definition of relational evaluation by including the need for some kind of approach to the interaction partner of the subsequently rejected person. This difference is important in the context of trusting others: situations in which participants make themselves vulnerable by asking for help or contact and in which rejection is possible require trusting others.

#### Trust and BPD

Trust is a fundamental requirement for social functioning since it is positively associated with cooperation, especially in situations with relatively strong conflicts of interest [22]. Although trust is a complex, multifaceted and heterogeneously defined concept, most definitions agree that trust comprises an individual’s willingness to make oneself vulnerable in situations of uncertainty in which a social counterpart may act in a way that benefits or harms the individual [23].

In BPD, most empirical studies on trust have assessed trustworthiness judgments for facial stimuli or trust behavior in economic exchange games such as the trust game. Most studies have found reduced trustworthiness appraisals and less trusting behavior in BPD patients (e.g. [24–31]; for reviews see: [3, 9, 26, 32]). These alterations have been shown to contribute to the perseverance of interpersonal difficulties reported by BPD patients, including a reduced sense of belonging indicated by high levels of loneliness (e.g. [4]).

Taken together with findings on the association between the severity of adverse childhood experiences (ACE) and lower interpersonal trust [33–35], the higher prevalence of self-reported ACE in patients with BPD [36, 37] points toward rejecting and invalidating experiences in early life as a potential underlying factor for lower interpersonal trust in BPD patients.

### Trustworthiness judgments and social rejection in BPD patients

Despite the many studies investigating social rejection and trustworthiness judgments in BPD patients, only a few studies have investigated their interrelationship in BPD patients. By asking participants to judge the intensity with which a facial stimulus expresses trustworthiness, studies have revealed an association between BPD features, rejection sensitivity and trustworthiness judgments: rejection sensitivity mediates the association between higher BPD features and lower trustworthiness ratings of neutral or untrustworthy facial stimuli [25, 28].

To our knowledge, no study has investigated how the experience of social rejection and acceptance affects trustworthiness judgments in BPD patients. Mayer et al. [38] proposed differentiating benevolence, ability and integrity as factors of trustworthiness appraisals providing unique perspectives explaining a major portion of trustworthiness judgments. They defined benevolence as ‘…the extent to which a trustee is believed to want to do good to the trustor, aside from an egocentric profit motive. Benevolence suggests that the trustee has some specific attachment to the trustor’ [38, p. 718]. The first impression of a trustee’s benevolence develops during the first contact and affects the perception of the interaction [39]. Thus, benevolence focuses on another person’s presumed attitude toward oneself and thus may be more relevant to an individual’s willingness to make oneself vulnerable in situations of potential rejection than judgments about another person’s ability in a given situation or their integrity as a relatively stable attribute. Therefore, benevolence is of particular interest in the context of interpersonal difficulties in BPD patients. However, no study so far investigated whether the difference in the perception of a social partners’ benevolence related to rejection and acceptance is altered in BPD patients compared to HCs.

### Inferring social causality and social judgments during social interactions in BPD patients

Inferring causality, or causal attributions, has been acknowledged as an influential factor in the development, growth or repair of trust [40]. Whether and how much the perception of the other person’s benevolence as a dimension of trustworthiness changes in the case of rejection or acceptance probably depends on which causes people attribute to the other person’s behavior [41]. If the reasons for rejection or acceptance are not known, the expectations a person has regarding the causality of social situations will influence whether they attribute the causes to internal, i.e., related to themselves, or external factors, i.e., related to the other person or the situation [42]. Positively associated with mental health is a “self-serving attributional bias”: causes for positive events are attributed more internally, and are temporally stable and independent of the specific situation (global) than are causes for negative events [43]. The opposite pattern has been associated with various mental disorders [44–46]. In line with these findings, it has been shown that individuals with BPD tend to attribute negative rather than positive events to (presumed) internal, stable and global causes [47]. Changes in attribution style have been linked to the processing of social rejection in BPD patients. Compared with HCs, patients with BPD allocated the reasons for being socially rejected in a virtual ball-tossing game to a greater extent to themselves and the hostile intentions of the co-player [48]. Consistent findings on BPD patients’ lower self-esteem and a stronger preference for information that confirms their own negative self-concept [2] point toward a potential process involved in the less beneficial attribution style in patients with BPD: to confirm their own negative self-concept, themselves are considered the cause of negative social interactions. In addition, it has been shown that a high severity of ACE is linked to reduced or even negative self-serving bias [43], suggesting that the often high severity of ACE in BPD patients is a potential underlying explanatory factor. Contrary, Berenson et al. [49] did not find differences between participants low versus high in BPD features in the majority of the most likely rated reasons for acceptance and rejection: both groups attributed rejection to negative qualities of the self and other or external reasons and acceptance to positive qualities of the self and other. Although there were group differences with regard to positive self and other attributions for rejection and negative self and other attributions for acceptance, these were generally rated as less likely by the participants. Interestingly, participants high in BPD features made more external attributions for acceptance compared to participants low in BPD features.

In sum, studies on the influence of alterations in causal attributions on the processing of social rejection and acceptance and trustworthiness judgments in BPD patients are sparse. Nevertheless, their findings point to alterations in inferring social causality that might interfere with the appraisal of social connectedness. A deeper understanding of the relevance of social attributions can be relevant for psychosocial interventions targeting maladaptive attributions to improve patients’ ability to cope with rejection and improve appreciation for social acceptance. However, to the best of our knowledge, no studies have investigated whether providing explanations for the behavior of others might influence social judgments in general or evaluations of another person’s benevolence in BPD patients. However, a beneficial effect on emotion regulation was shown in patients with major depressive disorder who reported a maladaptive attributional style. Loeffler et al. [50] demonstrated that providing an external explanation for social cues improved emotion regulation in these individuals: sadness related to negative cues and happiness related to positive cues decreased without significant differences compared to a HC group. So far, it has not been investigated whether providing external attributions has the same effects in BPD and how they affect the perception of the social partner. We assume that due to the combination of the tendency to verify, the often negative self-concept and unfavourable attributional style in BPD, the effect of provided external explanations might be reduced. Due to the tendency towards self-verification [2], an external justification of rejection could be less taken into account in processing. In contrast, an external justification of acceptance could confirm the assumption that one is merely accepted due to external circumstances, which is already more common in BPD when no explanation is explicitly given [49]. In consequence, an explicitly given external cause might have less impact. The tendency towards an unfavourable attributional style and self-verification of low self-esteem was also shown in MDD in the study by Löffler [50]. However, since the symptoms were remitted in over half of the patients in this study, this may have masked differences in the effects of provided external attributions.

### Aims of the present study

The present study investigated the effects of social rejection and acceptance on benevolence judgments in BPD patients. In addition, we were interested in whether the perception of an interaction partner’s benevolence could be influenced by providing an external, non-hostile explanation for their rejection or acceptance behavior. Based on previous research, we expected that (1) BPD patients assess an interaction partner in general as less benevolent than HCs, and that (2) being rejected or accepted and (3) receiving an external explanation for the interaction partner’s behavior affects the benevolence ratings of individuals with BPD less strongly than HCs. With regard to the influence of external explanations for the interaction partner’s behavior, we expect that 3a) the negative effect of rejection and 3b) the positive effect of acceptance on benevolence ratings are less strongly influenced by an external explanation in BPD patients than in HCs; that is, external explanations have a smaller positive effect on rejection and a smaller negative effect on acceptance in BPD patients than in HCs.

Additionally, we investigated whether the effects of our experimental manipulation are related to the individual’s general attributional style. We expect that 4) being rejected or accepted and 5) receiving an external explanation for the interaction partner’s behavior will affect the benevolence ratings more strongly for individuals with a greater tendency to attribute positive or negative events internally.

Finally, we explored whether the severity of adverse childhood experiences, self-esteem, rejection sensitivity and loneliness are related to the differences between the benevolence ratings induced by our experimental manipulations.

To investigate these research questions, we developed a virtual reality (VR) paradigm that simulates social acceptance and rejection by virtual characters in social interactions, during which the alleged conversation partner does or does not provide an external explanation of their behavior. Therefore, we used the advantages of VR approaches for studying complex social interactions, that is, the combination of a high ecological validity due to their immersiveness and experimental control in standardized social situations [51]. Additionally, compared to an imaginative vignette-based task, interactive VR paradigms are more emotionally engaging and immersive, and responses in VR predict real-life behavior as well as reactive and proactive motives beyond the hypothesized chosen response in the vignette task [52]. This might be an important advantage when investigating social rejection and acceptance. For example, it was shown that a VR environment with a stereo 3D view increases the emotional arousal triggered by the presentation compared to the corresponding 2D mode [53].

## Method

### Participants

Data collection took place between the 09th of May and the 2nd of November 2023. Participants had to be of legal age (18–65 years), understand and speak fluent German and, in the case of visual impairment, wear a visual aid. The general exclusion criteria were pregnancy, organic diseases of the brain, traumatic brain injuries, intellectual disability, epilepsy and significant current or past neurological diseases such as stroke, brain tumor or developmental disorders. Healthy subjects should neither be taking psychotropic drugs nor currently or previously have been suffering from a mental or neurological illness. For BPD patients, comorbid occurrence of the following mental disorders was an exclusion criterion for participation: schizophrenia, schizoaffective disorder, schizophrenia-like disorder, delusional disorder or bipolar I disorder. BPD patients were recruited from the wards and outpatient clinic of the Central Institute of Mental Health Mannheim (CIMH). The healthy control group was recruited via the CIMH website. A total of 35 participants with BPD were recruited, of whom three had to be excluded because they were not able to carry out the study by themselves (*n* = 2) or because they felt extremely uncomfortable during the measurement (*n* = 1), resulting in a patient sample of *n* = 32. A subsample of *n* = 32 healthy participants was selected from a larger sample of 76 participants (which was investigated in another study) to balance the groups for mean age, educational level and sex (see Table 1 for further demographic characteristics). The BPD diagnosis (meeting at least five of the nine DSM–IV criteria for BPD) was assessed by trained clinical psychologists using the International Personality Disorder Examination (IPDE; [54]), and further psychopathology was assessed via the structured clinical interview for DSM-IV (SKID-I; [55]). Four (12.5%) of the BPD patients were diagnosed with a harmful use or dependency syndrome of alcohol or cannabis, 25 (78.1%) with a depressive episode or recurrent depressive disorder, four (12.5%) with a phobic anxiety disorder, one (3.1%) with generalized anxiety disorder, and two (6.3%) with an obsessive-compulsive disorder. Furthermore, 12 (37.5%) patients were diagnosed with post-traumatic stress disorder, three (9.4%) with undifferentiated somatoform disorder, seven (21.9%) with an eating disorder, nine (28.2%) with attention deficit disorder (seven with and two without hyperactivity), one (3.1%) with tic disorder and three (9.4%) with a disorder of adult personality and behavior. The study was approved by the Research Ethics Board II of the Medical Faculty Mannheim of Heidelberg University.

To characterize the sample, we assessed BPD features and BPD and depressive psychopathology, individuals’ attributional style, self-esteem, rejection sensitivity, loneliness and the level of childhood traumatization.

#### Borderline personality features

Self-reported BPD features were assessed with the Borderline Scale from the Personality Assessment Inventory (PAI-BOR; [56], German version: [57]). Twenty-four items were answered on a 4-point Likert scale (0 ‘false’ – 3 ‘very true’; total score ranges from 0 to 72 with higher scores indicating more severe BPD features). In the present study, internal consistency was Cronbach’s α = 0.95 (BPD: α = 0.74; HCs: α = 0.68).

#### Borderline symptomatology

Self-reported severity of borderline symptoms within the last seven days was assessed with the short version of the Borderline Symptom List (BSL-23; [58]). Twenty-three items are answered on a 5-point Likert scale (0 ‘not at all’ – 4 ‘very strong’). The mean score ranges from 0 to 4 with higher scores indicating greater symptom severity. In the present study, internal consistency was Cronbach’s α = 0.98 (BPD: α = 0.95; HCs: α = 0.75).

#### Depressive symptoms

Self-reported depressive symptoms within the last two weeks were assessed using the Beck Depression Inventory ( BDI II; [59], german version: [60]). It contains 21 items rated on a list of four statements arranged according to increasing severity of specific symptoms (0–3). The sum score ranges from 0 to 63, with higher scores indicating greater symptom severity. In the present study, internal consistency was Cronbach’s α = 0.97 (BPD: α = 0.90; HCs: α = 0.81).

#### Attributional style

We assessed the participants’ tendency to attribute the causes of positive and negative events as internal or external with the German attribution style questionnaire for adults (ASF-E; [61]). The extent to which 8 positive and 8 negative events were attributed to external or internal causes was assessed on a 7-point Likert scale (1 ‘internal’ – 7 ‘external’) with two items each. Rating scores were summed separately for positive and negative events (range of sum scores 16–112). Higher scores indicate a stronger attribution to internal causes. In the present study, internal consistency of the internality subscale for positive events was Cronbach’s α = 0.88 (BPD: α = 0.84; HCs: α = 0.72) and the internality subscale for negative events was Cronbach’s α = 0.88 (BPD: α = 0.84; HCs: α = 0.79). Please note that more detailed analyses of the attributional style which includes, in addition to internality, also the dimensions of stability and globality are reported in a separate paper [62].

#### Self-esteem

Self-esteem was assessed using the Rosenberg Self Esteem Scale (RSES; [63], German version: [64]). It contains ten items that are rated on a 4-point Likert scale (0 ‘strongly disagree’ – 3 ‘strongly agree’). The total score ranges from 0 to 30 with higher scores indicating higher self-esteem. In the present study, internal consistency was Cronbach’s α = 0.95 (BPD: α = 0.81; HCs: α = 0.80).

#### Rejection sensitivity

Rejection sensitivity was measured with the Rejection Sensitivity Questionnaire for adults ( A-RSQ; [21], German version: [12]). For nine scenarios, participants rated their rejection concern (affective component) and expectancy (cognitive component) on a 6-point Likert scale (concern: 1 ‘very concerned’ – 6 ‘very unconcerned’, expectancy: 1 ‘very unlikely’ – 6 ‘very likely’). The scores were inverted, and for each item, the levels of concern and expectance were multiplied. The total score is the average of these nine multiplicative composites of the affective and cognitive ratings, ranging from 1 to 36, with greater scores indicating higher rejection sensitivity. In the present study, internal consistency was Cronbach’s α = 0.93 (BPD: α = 0.89; HCs: α = 0.83).

#### ACE severity

Retrospective self-reported ACE severity was assessed using the Childhood Trauma Questionnaire – Short Form (CTQ-SF; [65], German version: [66]). The scale consists of 28 items, answered on a 5-point Likert scale (1 ‘never true’ – 5 ‘very often true’). Three items are designed to capture the minimization and denial of problems. Five sub-scales with five items each assess childhood emotional, physical, and sexual abuse, as well as emotional and physical neglect. Subscales range from 5 to 25 with higher scores indicating more severe maltreatment. In the present study, internal consistency was Cronbach’s α = 0.95 (BPD: α = 0.93; HCs: Cronbach’s α = 0.91).

#### Loneliness

Frequency, intensity and duration of loneliness were assessed using a modified version of the UCLA loneliness scale (ULS; [67], German version: [68]). As proposed in Qualter et al. [69], participants state how often, intensely and long they feel a lack of companionship, feel left out, isolated from others and in tune with people around them on a 5-point Likert scale (frequency: 1 ‘never’ – 5 ‘very often’; intensity: 1 ‘not intense at all’ – 5 ‘very intense’; duration: 1 ‘not applicable’ – 5 ‘one month and longer’). Mean scores were calculated for the frequency, intensity and duration. These three facets of loneliness were combined into a composite score ranging from 1 to 5. In the present study, internal consistency was Cronbach’s α = 0.90 (BPD: α = 0.81 HCs: α = 0.86).

The sample characteristics are reported in Table 1. The BPD group reported higher levels of BPD features, BPD and depressive symptoms as well as higher levels of loneliness, rejection sensitivity, and ACE severity as well as lower levels of self-esteem. Participants of the BPD group attributed negative events to a greater extent and positive events to a lesser extent to themselves than did those in the healthy control group.

**Table 1:** Sample characteristics

	HC (*N* = 32)	BPD (*N* = 32)	test statistics	*p*-value
age	28.19 (8.14)	29.56 (13.07)	*t* = 0.51	0.616
sex assigned by birth	84.4% female	84.4% female	χ^2^ = 0	1.000
relationship	53.13% single	62.5% single	χ^2^ = 0.26	0.613
educational level^a^			Fisher’s exact test, two-sided	0.526
low	0%	9.38%		
intermediate	31.25%	31.25%		
high	68.75%	59.38%		
occupation			Fisher’s exact test, two-sided	< 0.001
unemployed	3.13%	34.38%		
student/pupil	53.13%	37.50%		
(self-) employed	43.75%	28.13%		
current treatment	0%	90.63%	Fisher’s exact test, two-sided	< 0.001
Borderline features (PAI-BOR)	19.59 (6.71)	52.09 (7.92)	*t* = 17.71, *d* = 4.43	< 0.001
Borderline symptoms (BSL23)	0.17 (0.16)	2.03 (0.95)	*t* = 10.81, *d* = 2.75	< 0.001
Depressive symptoms (BDI II)	4.69 (4.12)	33.66 (11.31)	*t* = 13.61, *d* = 3.40	< 0.001
Loneliness^b^ (ULS)	2.30 (0.61)	3.42 (0.62)	*t* = 7.17, *d* = 1.82	< 0.001
Self-esteem (RSQ)	25.09 (6.68)	10.50 (4.76)	*t* = -13.72, *d* = -3.43	< 0.001
Rejection sensitivity^b^ (RSQ)	6.21 (3.05)	15.90 (8.21)	*t* = 6.10, *d* = 1.56	< 0.001
Internal negative^b^ (ASF-E)	64.13 (10.41)	81.84 (14.81)	*t* = 5.48, *d* = 1.39	< 0.001
Internal positive^b^ (ASF-E)	75.75 (8.60)	56.68 (15.09)	*t* = -6.14, *d* = -1.57	< 0.001
ACE severity^b^ (CTQ-SF)	32.22 (9.34)	59.16 (18.75)	*t* = 7.18, *d* = 1.83	< 0.001

a = refers to the German school qualification system, low = less than 10 years of school education, intermediate = 10 years of school education, high = 12–13 years of school education. b = missing data of one BPD participant, *PAI-BOR *Personality Assessment Inventory Borderline Scale, *BSL23* Borderline Symptom List, *BDI II* Beck Depression Inventory, *ULS* UCLA Loneliness Scale, *RSQ* Rejection Sensitivity Questionnaire, *ASF-E* attributional style Questionnaire, *CTQ-SF* Childhood Trauma Questionnaire

### Procedure

After providing informed consent to participate in the study, a clinical interview was conducted to determine the current severity of BPD symptoms (BPD group) via the Zanarini Rating Scale for BPD (ZAN-BPD; [70]) or to rule out the presence or history of any mental illness (HC group) using the short diagnostic interview for psychological disorders (Mini-DIPS; [71]). Afterwards, participants completed the interaction paradigm in virtual reality and completed the questionnaires using the Research Electronic Data Capture tool (REDCap) [72, 73]. At the end, participants were debriefed and received a small compensation for participation.

### Virtual reality paradigm

After receiving initial instructions from the experimenter, and following a short familiarization phase with the setup, participants entered the VR environment while seated on an office chair. The VR was presented on an Oculus Quest 2 (Meta Platforms, Menlo Park, CA). Participants were asked to imagine that they had just moved to a city and were visiting the neighborhood festival that took place shortly afterwards. There, they got to know eight of their new neighbors during short conversations, one neighbor after another. During each of these eight conversations, they were asked to pose the same nine standardized questions, asking for advice or support. We experimentally manipulated the responses of the virtual characters in regard to (1) social acceptance and rejection (experimental factor ‘reaction’), and (2) whether the character offered an external explanation for their response or not (experimental factor ‘explanation’). Participants were asked to assess the character’s benevolence toward the participant directly following each response.

**Fig. 1:**
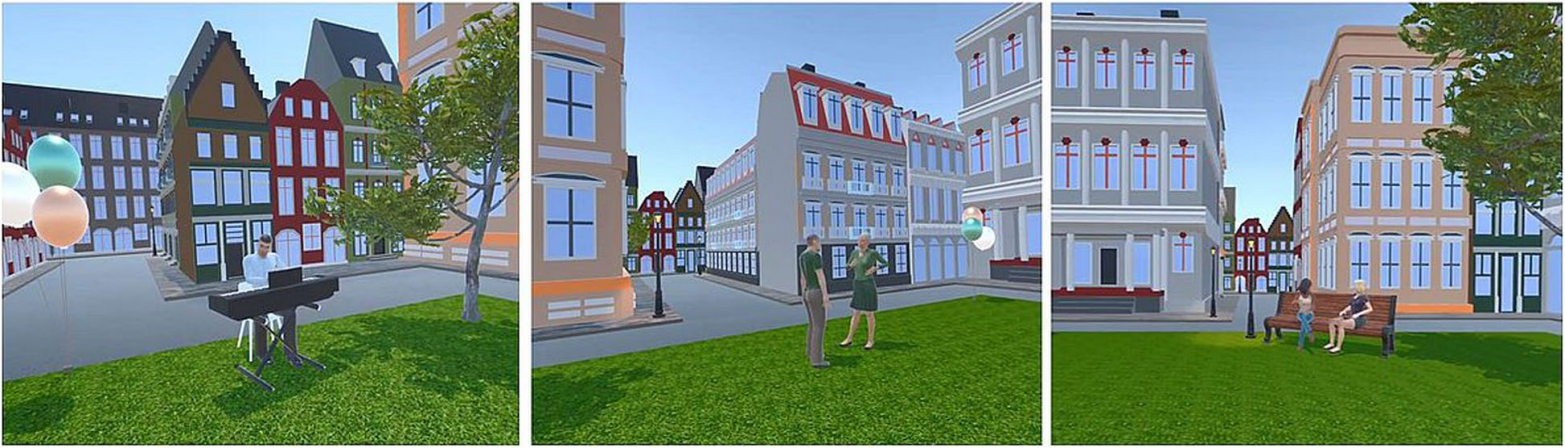
Impressions from the virtual environment

 The virtual interaction paradigm was developed in Unity software. The setting was a small, festively decorated city park with trees, surrounded by streets and houses. In the immediate vicinity, two virtual characters chatted on a bench, two others talked a few meters away and one male virtual character played the piano (Fig. 1). The background was filled with a low murmur of voices and relaxed piano music.

The neighbors were virtual characters generated via the Autodesk^®^ Character Generator. To control for possible confounders, all virtual characters were female and were presented in exactly the same size as the participants. The voices of the characters were generated using artificial intelligence (http://www.listnr.tech). All characters displayed neutral facial expressions and the same minimal arm movements during the whole social situation.

The questions were adapted from the RSQ. Since the original scenarios of the RSQ refer to people close to the participants such as romantic partners or parents, we adapted situations suitable for encounters with strangers. For example, participants were supposed to ask for information: “Would you tell me your personal insider tip for a cozy café or bar here in the neighborhood?”. For more details, see suppl. material table S1 and S2.

**Fig. 2:**
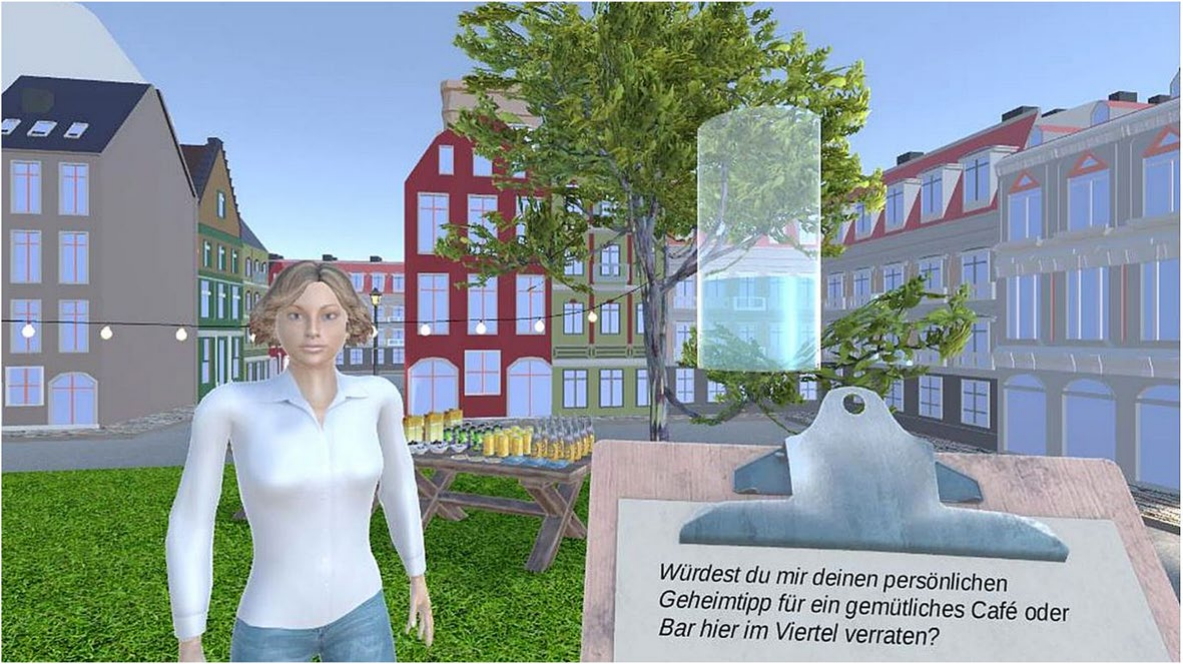
View of the VR paradigm with animated virtual character. The questions to be asked appeared on the clipboard, and the participants rated the perceived benevolence by changing the water level in the glass

 The participants were asked to read the questions displayed on a virtual clipboard out loud to enhance the feeling of presence. Afterwards, they were able to listen to the virtual character’s response by pressing a button on the controller. After listening to each answer, they indicated their perception of the virtual character’s benevolence by adjusting the water level in a virtual glass by pressing buttons on the controller. That is, when, after listening to an answer, participants felt that the person was indeed more benevolent toward them than previously thought, they were asked to increase the water level. In contrast, when, after an answer, they felt that the person was in fact less benevolent toward them than previously thought, they were asked to decrease the water level (Fig. 2). Participants could use the whole range from ‘empty’ to ‘full’ in 21 steps (coded as 0–20) when adjusting the water level on each occasion.

The four experimental conditions resulting from the combinations of the levels of the two experimental factors of the 2 × 2 design (factor reaction: acceptance and rejection; factor explanation: with and without an external cause) were presented in a pseudorandomized sequence. Therefore, the order of the four experimental conditions was balanced across the experiment, with each character rejecting or accepting the participants’ request in four to five out of their nine responses while providing an external explanation for four to five of their answers. The position of each of the four experimental conditions across the conversations with the characters as well as the changes in the four experimental conditions between consecutive trials were balanced across the experiment (for further details, see supplementary material Table S3). Whereas all participants underwent the same pseudorandomized sequence of responses for each virtual character, the order of the eight different characters varied and was counterbalanced across participants to control for possible differences between characters. Overall, 25% of all trials corresponded to each of the four experimental conditions.

#### Physical and social presence in virtual reality

To control for differences in the experience of the VR setting, we assessed physical and social presence in the virtual environment with two subscales of the Multimodal Presence Scale ( MPS; [74], german version: [75]). The physical sense of being there (physical presence) and the sense of being there with others (social presence) are assessed in two subscales of the MPS, with 5 items each answered on a 5-point Likert scale (1 (completely disagree) – 5 (strongly agree)). The subscale scores are averaged across the 5 items of the subscales (range: 1–5), with higher scores indicating stronger immersive experiences. Internal consistency in our sample for the physical presence subscale was Cronbach’s α = 0.78 (BPD: α = 0.80; HCs: α = 0.75) and for the social presence subscale α = 0.83 (BPD: α = 0.85; HCs: α = 0.80).

### Data analysis

We first compared the feelings of physical and social presence in virtual reality, as assessed with the MSP, between groups.

We then analyzed the outcome variable ‘perceived benevolence of a social partner’, as indicated by the water level in the virtual glass, which had been adjusted by participants after each question-answer interaction. We used linear mixed-effects (LME) models to analyze the differences in the effects of rejection and acceptance with and without an external explanation between BPD patients and HCs. Hereby, we investigated the “benevolence rating” (0–20) as the dependent variable and “group” (HC/BPD), “reaction” (rejection/acceptance) and “explanation” (no/yes) as the independent variables. For this purpose, we employed the *lmer* function of the *lme4* package [76] and the *lmerTest* package [77] to obtain *p* values in *R* 4.3.2 [78].

The model fit was obtained using the restricted maximum likelihood method (REML), BOBYQA optimizer and t-tests using Satterthwaite’s method. All interaction effects between these factors were included in the model to assess whether the combination of different levels of the factors had an effect on the dependent variable. Therefore, to enable the interpretation of both higher- and lower-order effects, we implemented effect coding via an orthogonal contrast scheme by the set_sum_contrasts() function as implemented in the *afex* package [79]. To account for the repeated measures, a by-subject random intercept was included. In addition, we also included random intercepts for the respective virtual character (8 character levels) and the specific question that participants had asked before receiving the character’s response (9 question levels).

All predictors were categorical and dichotomous (group: HC/BPD; reaction: rejection/acceptance; explanation: no/yes). We included a maximal random-effect structure, as recommended by Barr et al. [80]. Hence, we initially included by-subject random slopes for the within-subject factors reaction and explanation as well as their interaction term. However, the estimation of the maximal model did not converge. In the first step, we removed the covariance between the random intercept and random slopes, but still obtained singularity and convergence issues. Nevertheless, we still aimed to account for potential dependencies of reaction and explanation effects from all random factors, i.e., including character and question, as Scandola and Tidoni [81] demonstrated that removing the by-subject random slopes altogether would unreasonably strongly increase type I error (by increasing the estimated degrees of freedom and the risk of deflated errors for fixed effects estimates). For this reason, we decided to deviate from our preregistered approach and implemented a complex random intercept (CRI) structure, with separate random intercepts for each combination of reaction and explanation levels per participant (hence, nesting explanation and reaction within subject) instead of random slopes. Since the full-CRI model also resulted in a singular fit, we removed the CRI with the lowest variance as recommended by Scandola and Tidoni [81]. The final full-model specification was as follows (reported in *R / lme4* syntax here):

rating ~ group * reaction * explanation + (1 | subject) + (1|subject: reaction) + (1|subject: reaction: explanation) + (1|character) + (1|question).

Satterthwaite’s approximation method was applied for error estimation of *t* and *p* values for fixed effects coefficients [82]. The 95% confidence intervals (CIs) and *p*-values were computed using a Wald *t*-distribution approximation. For an investigation of interaction effects, post-hoc comparisons of least square means (i.e., a calculation of estimated marginal means per factor combination level) were performed using the *emmeans* package [83], and *p*-values were adjusted according to Benjamini and Hochberg [84].

To check the utility of the LME approach applied, we compared the full model with a null model via likelihood ratio tests. Assumptions (linearity, absence of collinearity, homoscedasticity, normality of residuals) were checked and met by the data. We chose a *p*-value threshold of less than 0.05 to indicate significant effects.

Exploratorily, we repeated the analyses described above with a new outcome variable derived from the original one, now using the *change* in evaluation, i.e., the difference score of the current and the previous benevolence evaluation, as the new dependent variable. As preregistered, in these analyses of change, we aimed to include sequence effects in the analyses, i.e., the two experimental conditions (reaction and explanation), as well as the previous benevolence rating of the preceding trial as influencing factors. However, this model appeared to be too extensive for our dataset, as it ran into singularity and convergence issues.

Although covering a broad range of 0–20 with small steps of 1, the benevolence rating is actually not a continuous scale, as modeled by linear mixed models, but rather an ordinal scale due to its restriction to integer values. Nevertheless, as Likert scales with a high number of levels, as in our case, can be considered continuous interval scales [85], we modeled as a continuous outcome variable to allow for a better interpretation of regression weights of predictors (fixed effects). To assess whether this approximation was justified, we conducted a consistency check and repeated our analyses with a cumulative link mixed-model using the *clmm* function of the *ordinal* package [86], which confirmed our findings. Details and results can be found in supplementary material Tables S7 and S8.

In a set of multiple linear regression analyses, we analyzed the correlations between psychological and biographical characteristics and the average responses for each experimental condition per participant. To investigate associations between alterations in benevolence perception and attributional style, we examined whether the tendency to attribute positive and negative events internally predicts the effects of the experimental factors of reaction and explanation (calculated as the average difference in benevolence ratings between acceptance and rejection trials and between trials with and without an external explanation). Furthermore, we explored whether the severity of ACE, the level of rejection sensitivity and self-esteem predict the effects of the experimental factors reaction and explanation. Finally, we explored whether the severity of ACE, rejection sensitivity, and self-esteem, together with the effects of the experimental factors reaction and explanation on benevolence, predict the level of loneliness.

After an initial screening of the data, we decided to exclude two BPD participants from the mixed-effects model analyses because the difference in their ratings for rejection and acceptance differed more than 3 SDs from the rest of the BPD group and substantially distorted the results. The analyzed sample for the mixed-effects model analyses thus consisted of 32 HCs and 30 BPD patients. The results of repeated mixed-effects model analyses with the complete sample can be found in supplementary material Table S6. The sample characteristics of the analyzed sample can be found in supplementary material Table S4.

Due to a lack of suitable effect size estimates from similar experimental setups, the recruitment process was guided by pragmatic considerations: over a pre-defined recruitment period (May – July 2023), all potential participants who presented with a confirmed BPD diagnosis (BPD group), or who had fulfilled the inclusion criteria for the healthy control group and the matching criteria (HC), were included into the sample. The data collection period was extended by three months in order to reach at least 30 participants per group. To obtain useful information for future studies, we conducted a post-hoc sensitivity analysis. For this purpose, we determined the minimum detectable effect (MDE) for the main interaction term in our main model, i.e., the 3-way interaction diagnosis × reaction × attribution. For this purpose, we used a numerical approach, simulating data based on the main model fitted to the empirical data. In ascending steps of 0.01, we changed the assigned effect size for the interaction term in the generative model, keeping all other model parameters equal to the empirically obtained values. Using the R package *simr* [87], we simulated *n* = 500 data sets for each effect size. MDE was defined as the first effect size for which sensitivity for obtaining a statistically significant result (i.e., the number of simulated data sets for which the refitted model detected a significant interaction effect) was above the threshold of 80%. Threshold for significance was set to 0.045, as recommended by the package author for z-test approximations of t-tests, to ensure conservative estimates.

### Pre-registration

The main hypotheses were pre-registered together with the design and planned analyses (https://aspredicted.org/9K3_QSN).

## Results

### Feelings of physical and social presence

The groups did not differ in terms of physical or social presence (physical: HC: *M* = 3.13, *SD* = 0.70; BPD: *M* = 3.06, *SD* = 0.82, *t* = -0.34, *p* = .738; social: HC: *M* = 2.48, *SD* = 0.71; BPD: *M* = 2.65, *SD* = 0.91, *t* = 0.85, *p* = .397). Compared to the “high quality representation of a social interaction partner” condition in the study of the German translation of the Multimodal Presence Scale by Volkmann et al. [75], the overall values across our whole sample were lower for physical presence (Volkmann sample: *M* = 3.54, *SD* = 0.71; our sample: *M* = 3.09, *SD* = 0.74; *t* = 3.178, *p* = .002, *d* = 0.646), but were comparably high for social presence (Volkmann sample: *M* = 2.61, *SD* = 1.04; our sample: *M* = 2.56, *SD* = 0.80; *t* = 0.284, *p* = .777, *d* = 0.055).

### Prediction of the level of the benevolence rating by group, reaction and explanation

**Fig. 3:**
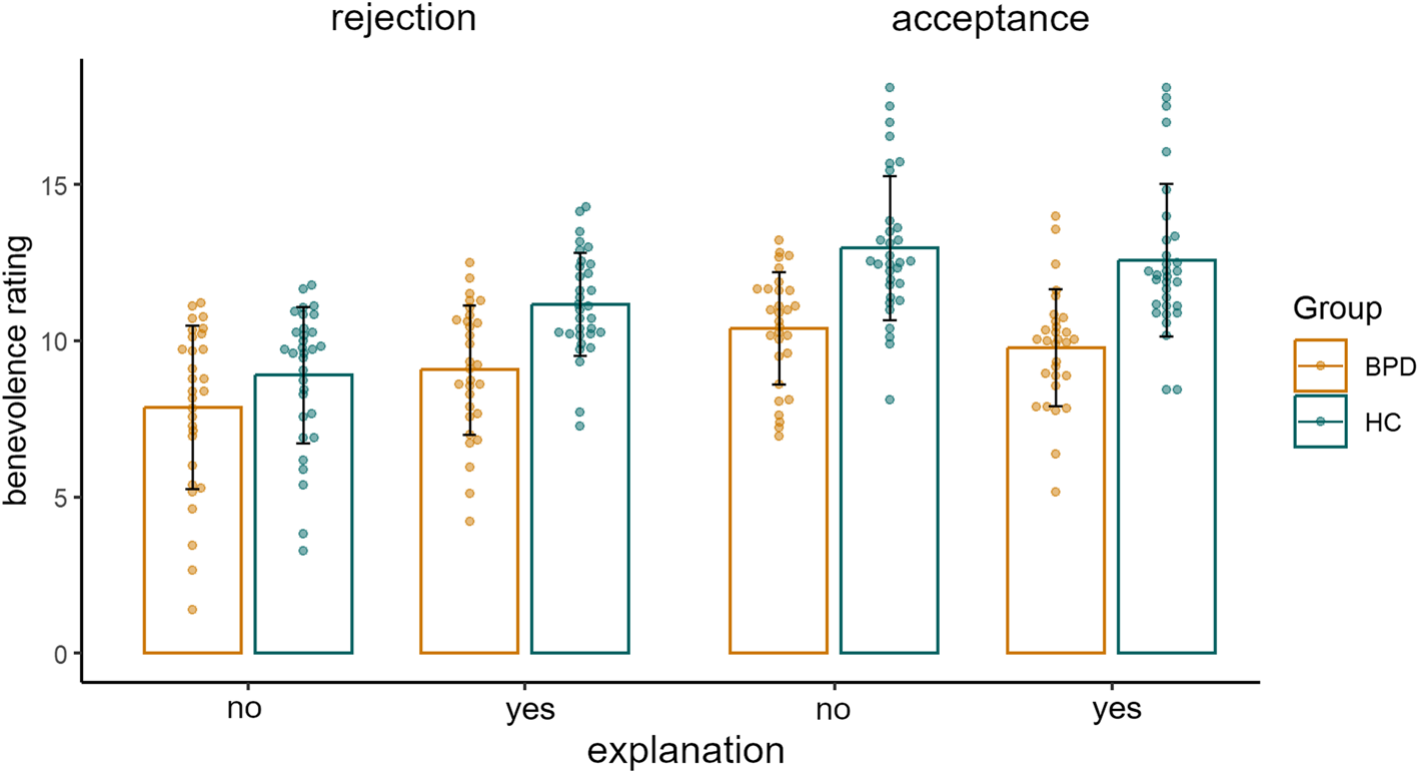
Mean benevolence ratings per group and condition. Dots represent mean ratings per person, and error bars indicate standard deviations

 The means and standard deviations of the benevolence ratings according to group and condition are displayed in Fig. 3. See also suppl. material Table S5.

We fitted a linear mixed model to predict benevolence ratings with the factors group, reaction and offer of an explanation as well as their interaction terms. The model included the following reduced CRI structure: (1|subject) + (1|subject: reaction) + (1|VP: reaction: explanation) + (1|character) + (1|question). The model’s total explanatory power is substantial (conditional *R*^*2*^ = 0.519). The proportion of explained variance related to the fixed effects alone (marginal *R*^*2*^) was 17.2%, indicating that 34.7% of the variance in benevolence ratings was explained by the CRIs. Model comparison revealed a significantly better model fit compared to the null model without any fixed effects (*χ*^*2*^ (7) = 128.89, *p* < .001). For further details, see Table 2 and supplementary material Table S9.

The model’s fixed intercept, corresponding to the grand mean, is at 10.35. Benevolence ratings were 2.12 points lower for participants in the BPD group (main effect of group). An investigation of the significant interaction effect group × reaction via post-hoc comparison of marginal means revealed that the difference between benevolence ratings of acceptance versus rejection was smaller in the BPD group than in the HC group (HC: *Z* = -8.29, *p*_*FDR*_ < 0.001; BPD: *Z* = -4.77, *p*_*FDR*_ < 0.001).

In addition, the difference in the benevolence rating between evaluations with and without an explanation was smaller in the BPD group than in the HC group (interaction group × explanation). Post hoc analyses of the model’s marginal means revealed that providing an external explanation increased benevolence ratings in HCs, while this effect was only present at trend level for BPD patients (HC: *Z* = -5.14, *p*_*FDR*_ < 0.001; BPD: *Z* = -1.82, *p*_*FDR*_ = 0.069, Fig. 3).

The three-way interaction of group × reaction × explanation was not significant. However, the mean benevolence ratings suggest that the stronger effect of explanation in the HC group can be attributed primarily to the rejection condition. For further details, see Fig. 3 and Table S5.

Furthermore, analyses revealed a significant interaction effect of reaction and explanation; however, there was no differential effect for either group. The effect of providing an explanation differed significantly between both types of reactions (interaction reaction × explanation). Post-hoc analyses of the model’s marginal means revealed that benevolence ratings after rejection trials were greater if an explanation was given (*Z* = -7.26, *p*_*FDR*_ < 0.001), while providing an external explanation did not influence benevolence ratings in acceptance trials (*Z* = 0.47, *p*_*FDR*_ = 0.641, Fig. 3).

Due to the higher-order interaction effect, the main effects of reaction and explanation are of limited interpretability.

**Table 2:** Fixed effects for the linear mixed model predicting benevolence ratings

	Estimate	95% CI	Std. Error	df	t	*p*
Intercept	10.35	9.43, 11.27	0.47	13.97	21.95	< 0.001
Group	1.06	0.63, 1.50	0.22	60.01	4.80	< 0.001
Reaction	-1.10	-1.33, -0.86	0.12	60.20	-9.19	< 0.001
Explanation	-0.35	-0.49, -0.21	0.07	120.35	-4.88	< 0.001
Reaction × Explanation	-0.40	-0.55, -0.25	0.07	134.90	-5.39	< 0.001
Group × Reaction	-0.28	-0.51, -0.05	0.12	60.01	-2.34	0.023
Group × Explanation	-0.16	-0.30, -0.02	0.07	119.36	-2.27	0.025
Group × Reaction × Explanation	-0.10	-0.24, 0.04	0.07	119.36	-1.44	0.154

The analysis relies on orthogonal sum-to-zero contrasts

For technical reasons (requirements by the *simr* package), as well as for better interpretability of the three-way interaction effect, the post-hoc determination of MDE size was based on a refitted model that employed treatment contrasts instead of orthogonal contrasts. The actual effect size of the interaction retrieved from our empirical data with treatment contrasts (group = BPD, reaction = acceptance, explanation = yes) was 0.83. Our simulation revealed an MDE of 1.61 as necessary to be detected with 80% sensitivity. We can thus estimate that, *ceteris paribus*, a potentially present interaction effect would have had to be twice as large as it actually was, in order to be detected reliably in our sample.

### Association of benevolence ratings with attributional style

In both groups, the effects of the two experimental factors were not correlated with the individual’s attributional style. For further details, see supplementary material Tables S10, S11, S12 and S13.

### Associations of benevolence ratings with the severity of ACE, rejection sensitivity, self-esteem and loneliness

In both groups, the effects of the two experimental factors were not correlated with the severity of ACE, rejection sensitivity, self-esteem or loneliness. For further details, see supplementary material Tables S10, S11, S14, S15 and S16.

## Discussion

In the present study, we investigated differences in the influence of external explanations for rejection and acceptance on the benevolence perception of a virtual social partner between patients with BPD and HCs. In addition, we examined the associations of benevolence perceptions with an individual’s attributional style, rejection sensitivity, self-esteem, ACE severity and loneliness. Overall, our findings indicate that overall, the level of benevolence ratings was lower in the BPD group than in the HC group. Across both groups, a social partner was perceived as less benevolent following rejection than following acceptance. The difference in benevolence ratings between BPD patients and healthy control participants was greater following acceptance than following rejection. An external explanation for a rejection mitigated the negative effect of rejection. Independent of acceptance and rejection, an external explanation was associated with a higher level of benevolence only in the HC group. Our findings revealed no unique prediction of the effects of our experimental conditions by attributional style, severity of ACE, rejection sensitivity or self-esteem. Similarly, benevolence ratings did not predict loneliness. We will briefly discuss each of these findings in the following.

### Benevolence perception is reduced in BPD patients

Benevolence is considered to be one dimension of trustworthiness [38]. Thus, our finding that BPD patients have lower overall levels of perceived benevolence in a social partner is consistent with our first hypothesis and with numerous studies of altered trustworthiness ratings and trust behavior in patients diagnosed with BPD [24–31]. Our results extend previous findings in that the less positive perception of a social partner in BPD refers not only to the characteristics of the other person, but also directly to the presumed attitude of the social partner toward oneself. It can be reasonably assumed that a generalized reduction in the perception of others’ benevolence contributes to the interpersonal difficulties commonly observed in BPD patients. It can be argued that this may have the effect of reducing the incentive to invest in social contacts and may also make it more difficult to resolve conflictual social situations. If this is also the case in already established, closer social relationships, the reduced sense of others desiring one’s well-being may also contribute to a diminished capacity for connection, which in turn may contribute to the elevated level of loneliness observed in BPD patients. In our BPD subsample, a lower overall level of perceived benevolence was only marginally associated with higher levels of loneliness (*r* = − .31, *p* = .098, for further details see suppl. Table S10). To further investigate the association between benevolence and loneliness in BPD patients, studies with larger samples are needed. Moreover, future research might investigate whether the lower benevolence judgments of a social partner in patients with BPD compared to HCs refer to a general negative bias when judging the trustworthiness of others or whether they relate rather specifically to situations involving interactions with oneself. In the latter case, benevolence judgments would be altered in the context of self-referential processing, that is, when judging another person’s benevolence toward the person oneself, and might be related to an individual’s self-esteem.

### Effect of acceptance and rejection on benevolence perception in BPD patients compared with HCs

Benevolence was judged to be greater following acceptance than rejection. This indicates that our experimental manipulation was successful in both groups. The findings of other studies suggest that a (emotional) high sensitivity toward potential rejection contributes to lower interpersonal trust in BPD patients [25, 28]. In our study, in contrast, the differential effect between BPD patients and controls was more prominent for the opposite kind of reaction, namely, for acceptance: our findings revealed a stronger group difference between BPD patients and HCs for acceptance trials than for rejection trials, which was due to a greater increase in the level of benevolence ratings after acceptance in the HC group than in the BPD group. This confirmed our second hypothesis. This extends findings of prior studies reporting a general unspecific negative bias toward perceived participation in the cyberball paradigm [16, 88, 89] and altered cognitive and behavioral responses to social acceptance in BPD [90] in two ways. First, our paradigm demonstrated that these differences are also evident in the context of rejection and acceptance in a narrow sense – that is, when an interaction partner may either decline or comply with an explicit request made by oneself – in contrast to previous studies that induced social exclusion or social evaluation instead of actual rejection. Second, our findings offer a potential explanation for why individuals with BPD find it challenging to adjust their expectations of social acceptance in the wake of positive feedback [90]: it has a less positive impact on their perception of their social partner’s attitude toward themselves. Therefore, our data underscore the importance that the focus of therapeutic approaches for the treatment of interpersonal difficulties in BPD should not be limited to coping with rejection but should also include a strengthening of the perception and enjoyment of being accepted. Furthermore, prior findings suggest altered social norms in BPD in form of higher expectations regarding inclusion and fairness [17, 91]. In this context, the lower effect of acceptance could also be explained by altered expectations and an idealized need for belonging in social relationships in BPD. If this is confirmed in future research, interventions that support patients in better balancing the discrepancy between idealized expectations and experience could be helpful.

### Effect of external explanations on benevolence perception in BPD patients compared with HCs

As expected, an external explanation for rejection mitigated its negative effect on benevolence judgments. In contrast, external explanations did not influence benevolence perception after acceptance. While this effect was observed across all participants, our analyses revealed differences between groups in the effect of an explanation independent of the accepting or rejecting behavior of the social partner. In line with our third hypothesis, analyses revealed a positive effect of explanation on benevolence only in the HC group, while this effect was evident in the BPD group only at a trend level.

Loeffler et al. [50] found that patients with major depressive disorder (MDD) and HCs were similarly able to regulate positive and negative emotions with instructed causal attributions. In contrast, our data suggest that external explanations had less influence on attributing benevolence to a social interaction partner in BPD patients than in HCs. While this might point to a differential impact of providing an explanation for events in MDD and BPD, differences between studies might also be caused by differences in the target constructs. While Loeffler et al. [50] investigated the effects of instructed imagination of being the cause of depicted happiness or sadness during the presentation of emotional face stimuli on feelings of happiness and sadness, our paradigm not only targeted a more complex social judgment (namely, benevolence), but also arguably introduced an ecologically more valid and more complex context with the VR interaction situation. Further research is needed to investigate whether the effects of providing explanations differ between emotional states and social behaviors and between different types of socio-affective judgments and different disorders. Nevertheless, our results are in line with previous findings on the difficulties of integrating of multiple perspectives in BPD [92], and therefore provide further support for the importance of strengthening the mentalizing ability of BPD patients as part of therapy. It is important to note that further research is needed to first investigate in which step of the process (e.g. attentional focus, processing or evaluation of information) changes to develop targeted interventions. Although providing an external explanation did not have a significant effect on benevolence ratings in BPD patients, the effect was evident at a trend level. Together with findings that attributional retraining is beneficial for the attributional style of individuals with low self-esteem [93], this suggest that techniques such as attribution retraining and cognitive reappraisal may be at least partially helpful for these patients in coping with rejection. In addition, several psychotherapeutic approaches, such as cognitive-behavioral therapy and mentalization -based treatment, aim to improve the ability to apply helpful attributions to facilitate emotion regulation.

Contrary to our hypotheses 3a and 3b, we did not find a differential effect of providing an explanation for rejection and acceptance between groups. It is important to note that sensitivity analysis indicated that a potentially present interaction effect would have had to be twice as large as it actually was, in order to be detected reliably in our sample. Regarding rejection, the mean benevolence ratings support the assumed smaller positive effect of an external explanation in BPD patients than in HCs. Regarding acceptance, the negative effect of explanation is indicated by mean benevolence ratings in both groups but did not reach significance. However, our data did not support the expected smaller negative effect of explanation on acceptance in BPD compared to HC.

### Consequences and predictors of altered benevolence differentiation

The alterations in the differences in benevolence ratings between rejection and acceptance, as well as between the effects of providing an external explanation for the interaction partner’s behavior, were not explained by attributional style, ACE severity, rejection sensitivity or self-esteem in BPD patients. More research is needed to identify intrapersonal predictors of benevolence perception. Similarly, self-esteem as a more intra- than interpersonal-related factor seems to be more relevant for the perception of loneliness than alterations in differentiating between the reactions of social partners in BPD, supporting prior findings on the potentially mediating role of low self-esteem in the association between ACE severity and loneliness [94, 95].

### Strengths and limitations

To our knowledge, our study is the first to experimentally induce rejection and acceptance in a similar way to how rejection is constructed in the literature: by creating a situation in which participants make themselves vulnerable by asking for help or contact. The groups did not differ in the level of physical or social presence experienced in the VR paradigm. Compared to the “high quality representation of a social interaction partner” condition in the study of the German translation of the Multimodal Presence Scale [75], the overall values across our whole sample were lower for physical presence, but comparably high for social presence. Therefore, differences between groups were not due to differences in immersion or the suitability of VR paradigms for investigating alterations in social cognition in BPD patients. Although some of the BPD patients reported regular dissociative experiences in their everyday life, they did not report the examination using the VR headset as unpleasant or dissociation-triggering.

Some limitations need to be addressed. The first refers to our sample: due to the overrepresentation of female BPD patients in the healthcare system, the generalizability of our findings to men is restricted. Together with findings indicating that rejection is experienced differently by male and female participants [96, 97], these findings emphasize the need for more research to investigate differences in the experience of rejection and acceptance between male and female BPD patients. The small sample size in the sub-group regression analyses could have concealed possible associations and did not allow us to test for interaction effects of the experimental factors and traits. In addition, the investigated sample size was below the “corridor of stability” for correlation analyses [98], emphasizing the need to replicate our findings in larger samples.

With regard to our experimental design, a discrepancy between the focus of the RSQ and our adapted items based on the RSQ is apparent: while the RSQ describes situations of potentially high emotional relevance with close others, we investigated experiences in first encounters with strangers. This might explain why rejection sensitivity as assessed with the RSQ, did not predict benevolence perception in our paradigm. Future VR research might utilize 3D scans of close others to investigate the experience of rejection and acceptance in close relationships. In addition, the non-significant effect of an external explanation for acceptance can be interpreted in two ways: One explanation could be that for the perception of benevolence after being accepted by a stranger, the underlying motivation is not relevant. An alternative explanation would be that our wording of the external explanations for acceptance did not emphasize strongly enough that the reason for the positive reaction was not related to the participant. In this context, it is important to note that the items used were not previously rated by independent coders and subsequent studies should evaluate in independent sample how people would attribute the locus of control. Larger samples and potentially a stronger emphasis on externalities in the explanations of acceptance might be necessary to further investigate the potential effect of an explanation for acceptance. In addition, further directions for future studies include investigating the influence of internal explanations, as well as the influence of differences in the dimensions of globality and the stability of explanations.

Finally, we only used a benevolence scale to record how the test subjects reacted to the experimental situation. Further studies might benefit from using more proximal measures, such as the closeness or feeling of being rejected, to differentiate between changes related to the current interaction and more trait-like judgments such as benevolence as one facet of trustworthiness. More detailed investigations are therefore necessary to examine whether the external explanations provided actually led to different ratings via a change in the locus of control of the attribution, or whether the longer explanations simply had the effect of the interaction partners appearing friendlier as they were “taking their time”.

## Conclusion

This study is the first to utilize virtual reality to examine the impact of social rejection and acceptance, in conjunction with external explanations, on perceptions of benevolence in individuals with BPD. Our data indicate that acceptance and provided external explanations for rejection might have a less positive influence on the perception of a social partner’s benevolence in BPD patients than in HCs. Since we did not find any associations between the effects of the experimental conditions and attributional style, childhood trauma, rejection sensitivity, self-esteem or loneliness, more research is needed to identify predictors of benevolence perception and which steps of social information processing are altered. The therapeutic implications for the treatment of interpersonal difficulties in BPD patients include strengthening the perception and enjoyment of being accepted as well as improving the mentalizing ability of BPD patients. However, due to the described limitations, our findings have to be interpreted with care and have to be replicated in independent studies with larger samples.

## Supplementary Information

Supplementary Material 1.

## Data Availability

According to European law (GDPR), data containing potentially identifying or sensitive patient information are restricted; our data involving clinical participants are not freely available in the manuscript, supplemental files, or in a public repository. Data access can be requested on reasonable demand via AS.

## Abbreviations

ACE: adverse childhood experiences
ASF-E: Attributional Style Questionnaire
BPD: borderline personality disorder
BDI II: Beck Depression Inventory
BSL23: Borderline Symptom List
CTQ-SF: Childhood Trauma Questionnaire
HC: healthy control
PAI-BOR: Personality Assessment Inventory Borderline Scale
RSQ: Rejection Sensitivity Questionnaire
ULS: UCLA Loneliness Scale
VR: virtual reality
