# Supplementary material for: You say it‘s not me: the influence of offering external explanations of rejection and acceptance behavior on the perception of benevolence in borderline personality disorder
Source: Borderline Personal Disord Emot Dysregul. 2024 Nov 25;11:29. doi: 10.1186/s40479-024-00275-y (PMC11587614; doi:10.1186/s40479-024-00275-y)
Supplement: Supplementary file 1 — Supplementary Material 1. [file 40479_2024_275_MOESM1_ESM.pdf]

Supplemetary Material

You say it‘s not me? The influence of offering external explanations of rejection and acceptance behavior on perception of benevolence in borderline personality disorder

Schulze, A[1], Rommelfanger, B[1], Schendel, E[1], Kammler-Sücker, K[2,3]*, Lis, S[1,4]*

[1]Department of Clinical Psychology, Central Institute of Mental Health, Medical Faculty Mannheim, University of Heidelberg, Mannheim, Germany

[2]Center for Innovative Psychiatric and Psychotherapeutic Research (CIPP), Central Institute of Mental Health, Medical Faculty Mannheim, University of Heidelberg, Mannheim, Germany

[3]Institute of Cognitive and Clinical Neuroscience, Central Institute of Mental Health, Medical Faculty Mannheim, University of Heidelberg, Mannheim, Germany

[4]Department of Psychosomatic Medicine and Psychotherapy, Central Institute of Mental Health, Medical Faculty Mannheim, University of Heidelberg, Mannheim, Germany

*contributed equally

Table S1

Stimulus Material - Wording of the dialogues

		Reaction	
		Acceptance	Rejection
Scenario 1		Hello, would you mind telling me something about the history of the district that is celebrated here?	
Explanation	No	Yes, I can tell you a few sentences later. (VC1)<br>Yes, I can tell you a few sentences later. (VC5)	No, ask someone else (VC2)<br>No, ask someone else. (VC6)
	Yes	Yes, I can tell you a few sentences later, because I am very interested in it. (VC3)<br>Yes, I can tell you a few sentences later, I have a few minutes to spare anyway (VC7)	No, ask someone else, I can't think of anything right now. (VC4)<br>No, ask someone else, I can't think of anything right now. (VC8)
Scenario 2		Would you tell me your personal insider tip for a cozy café or bar here in the neighborhood?	
Explanation	No	Yes, the Café Zimt, which is just around the corner. (VC4)<br>Yes, the Bar Berlino, it's just around the corner. (VC8)	No, I won't tell you. (VC3)<br>No, I won't tell you. (VC7)
	Yes	Yes, the Café Zimt, it's just around the corner, I always tell everyone about it so that the café stays open. (VC5)<br>Yes, the Bar Berlino, it's just around the corner, I always tell everyone who asks me about it. (VC6)	No, I won't tell you, because I don't have a favorite bar. (VC1)<br>No, I won't tell you, because I don't have a favorite café. (VC2)
Scenario 3		I heard there is a neighborhood association here. Would you show me where the meetings are held?	
Explanation	No	Yes, that's in the red house right behind me. (VC7)<br>Yes, that's in the red house right behind me. (VC8)	No, that's not possible. (VC3)<br>No, that's not possible. (VC4)
	Yes	Yes, it's in the red house right behind me, we're still looking for people to take on tasks anyway. (VC1)<br>Yes, that's in the red house right behind me, I wanted to go there anyway. (VC5)	No, that's not possible, I've never been there. (VC2)<br>No, that's not possible, I've never been there. (VC6)
Scenario 4		I still have to decide for my electricity contract whether I want to stay with the basic supplier or switch, can you give me your advice?	
Explanation	No	Yes, the municipal utilities are the most reliable. (VC1)<br>Yes, the municipal utilities are the most reliable. (VC3)	No, I can’t. (VC6)<br>No, I can’t. (VC8)
	Yes	Yes, the municipal utilities are the most reliable, I was just researching that yesterday. (VC2)<br>Yes, the municipal utilities are the most reliable, I know someone who has been working there for a long time. (VC7)	No, I can't, because I'm not really familiar with that subject. (VC4)<br>No, I can't. I'm also currently searching because I'm unhappy with mine. (VC5)

Scenario 5		I forgot to take my cell phone with me and have an important call to make later, could I borrow yours later?	
Explanation	No	Yes, no problem. (VC2)<br>Yes, no problem. (VC4)	No, that's not possible. (VC1)<br>No, that's not possible. (VC5)
	Yes	Yes, no problem, I still have a lot of free minutes left this month anyway, it would be a shame if they expired. (VC6)<br>Yes, no problem, I always have unlimited free minutes. (VC8)	No, that's not possible, I don't have one with me at the moment. (VC3)<br>No, that's not possible, the battery in mine has just run out. (VC7)
Scenario 6		I have to take the bus later, but I don't have any change for the ticket, could you lend me €3?	
Explanation	No	Yes, I'll lend you the money. (VC4)<br>Yes, I'll lend you the money. (VC5)	No, that's not possible. (VC1)<br>No, that's not possible. (VC7)
	Yes	Yes, I'll lend you the money, I always have a few euros left over. (VC6)<br>Yes, I'll lend you the money, I've just found a fiver anyway. (VC8)	No, that's not possible because I'm always short of money. (VC2)<br>No, that's not possible because I don't have any money with me at the moment. (VC3)
Scenario 7		I still have to put up my curtains, can you lend me your drill soon?	
Explanation	No	Yes, you can have it for a few days. (VC2)<br>Yes, you can have it for a few days. (VC6)	No, that's not possible. (VC7)<br>No, that's not possible. (VC8)
	Yes	Yes, you can have it for a few days, I don't have to drill anything for a while. (VC3)<br>Yes, you can have it for a few days, it's just lying around at my place anyway. (VC4)	No, that's not possible because I don't have one. (VC1)<br>No, that's not possible because I need it myself at the moment. (VC5)
Scenario 8		For next Saturday afternoon I'm planning a coffee date to get to know some neighbours, would you like to join?	
Explanation	No	Yes, I'll join you. (VC2)<br>Yes, I'll join you. (VC3)	No, I won't be there. (VC5)<br>No, I won't be there. (VC6)
	Yes	Yes, I'll join you, I don't have any plans yet. (VC1)<br>Yes, I'll join you, I've just got the day off. (VC7)	No, I won't be there - I always have to work on Saturday afternoons. (VC4)<br>No, I won't be there, I already have an appointment. (VC8)
Scenario 9		Thanks for the nice conversation, shall we sit down and have a chat?	
Explanation	No	Yes, we can do that. (VC2)<br>Yes, we can do that. (VC5)	No, that's not possible. (VC1)<br>No, that's not possible. (VC6)
	Yes	Yes, we can do that, I still have time to pass until my appointment anyway. (VC3)<br>Yes, we can do that, I have nothing else to do at the moment anyway. (VC7)	No, that's not possible, I'm tired and always have to get up early in the morning. (VC4)<br>No, that's not possible, because I have to go straight on. (VC8)

Note: VC: virtual character

Table S2

Stimulus Material – original German wording of the dialogues

		Reaction	
		Acceptance	Rejection
Scenario 1		Hallo, magst du mir nachher etwas zu der Geschichte von dem Stadtteil erzählen, der hier gefeiert wird?	
Explanation	No	Ja, da kann ich später ein paar Sätze zu erzählen. (VC1)<br>Ja, da kann ich später ein paar Sätze zu erzählen. (VC5)	Nein, da frag mal jemand anderes. (VC2)<br>Nein, da frag mal jemand anderes. (VC6)
	Yes	Ja, da kann ich später ein paar Sätze zu erzählen, weil ich mich sehr dafür interessiere. (VC3)<br>Ja, da kann ich später ein paar Sätze zu erzählen, ich habe gerade eh ein paar Minuten übrig (VC7)	Nein, da frag mal jemand anderes, da fällt mir gerade nichts zu ein. (VC4)<br>Nein, da frag mal jemand anderes, da fällt mir gerade nichts zu ein. (VC8)
Scenario 2		Würdest du mir deinen persönlichen Geheimtipp für ein gemütliches Café oder eine Bar hier im Viertel verraten?	
Explanation	No	Ja, das Café Zimt, das ist hier direkt um die Ecke. (VC4)<br>Ja, die Bar Berlino, die ist hier direkt um die Ecke. (VC8)	Nein, den verrate ich nicht. (VC3)<br>Nein, den verrate ich nicht. (VC7)
	Yes	Ja, das Café Zimt, das ist hier direkt um die Ecke, da erzähle ich immer allen von, damit das Café bestehen bleibt. (VC5)<br>Ja, die Bar Berlino, die ist hier direkt um die Ecke, da erzähle ich immer allen von, die mich fragen. (VC6)	Nein, den verrate ich nicht, denn ich habe keine Lieblingsbar. (VC1)<br>Nein, den verrate ich nicht, denn ich habe kein Lieblingscafé. (VC2)
Scenario 3		Ich habe gehört es gibt hier einen Nachbarschaftsverein. Würdest du mir zeigen, wo die Treffen stattfinden?	
Explanation	No	Ja, das ist in dem roten Haus direkt hinter mir. (VC7)<br>Ja, das ist in dem roten Haus direkt hinter mir. (VC8)	Nein, das geht nicht. (VC3)<br>Nein, das geht nicht. (VC4)
	Yes	Ja, das ist in dem roten Haus direkt hinter mir, wir suchen eh noch Leute, die Aufgaben übernehmen. (VC1)<br>Ja, das ist in dem roten Haus direkt hinter mir, da wollte ich gleich eh noch hin. (VC5)	Nein, das geht nicht, ich war da noch nie. (VC2)<br>Nein, das geht nicht, ich war da noch nie. (VC6)
Scenario 4		Ich muss mich für meinen Stromvertrag noch entscheiden, ob ich beim Grundversorger bleiben oder lieber wechseln will, kannst du mir da eine Einschätzung zu geben?	
Explanation	No	Ja, die Stadtwerke sind am zuverlässigsten. (VC1)<br>Ja, die Stadtwerke sind am zuverlässigsten. (VC3)	Nein, das geht nicht. (VC6)<br>Nein, das geht nicht. (VC8)
	Yes	Ja, die Stadtwerke sind am zuverlässigsten, da habe ich gestern erst zu recherchiert. (VC2)	Nein, das geht nicht, denn ich kenne mich mit sowas nicht wirklich aus. (VC4)

		Ja, die Stadtwerke sind am zuverlässigsten, da kenn ich jemanden, der da schon lange arbeitet. (VC7)	Nein, das geht nicht, ich bin auch gerade am Suchen, da ich mit meinem unzufrieden bin. (VC5)
Scenario 5		Ich habe vergessen mein Handy mitzunehmen und muss nachher einen wichtigen Anruf erledigen, könnte ich mir später deins leihen?	
Explanation	No	Ja, kein Problem. (VC2)<br>Ja, kein Problem. (VC4)	Nein, das geht nicht. (VC1)<br>Nein, das geht nicht. (VC5)
	Yes	Ja, kein Problem, ich habe diesen Monat eh noch viele Freiminuten übrig, es wäre schade, wenn die verfallen. (VC6)<br>Ja, kein Problem, ich habe immer unbegrenzt viele Freiminuten. (VC8)	Nein, das geht nicht, ich habe gerade keins dabei. (VC3)<br>Nein, das geht nicht, der Akku von meinem ist gerade leer. (VC7)
Scenario 6		Ich muss später mit dem Bus fahren, habe aber kein Kleingeld mehr für das Ticket, könntest du mir 3€ leihen?	
Explanation	No	Ja, ich leihe dir das Geld. (VC4)<br>Ja, ich leihe dir das Geld. (VC5)	Nein, das geht nicht. (VC1)<br>Nein, das geht nicht. (VC7)
	Yes	Ja, ich leihe dir das Geld, ein paar Euro habe ich immer übrig. (VC6)<br>Ja, ich leihe dir das Geld, ich habe gerade eh einen Fünfer gefunden. (VC8)	Nein, das geht nicht, weil ich immer knapp bei Kasse bin. (VC2)<br>Nein, das geht nicht, weil ich gerade kein Geld dabei habe. (VC3)
Scenario 7		Ich muss noch meine Vorhänge anbringen, kannst du mir demnächst deine Bohrmaschine leihen?	
Explanation	No	Ja, die kannst du für ein paar Tage haben. (VC2)<br>Ja, die kannst du für ein paar Tage haben. (VC6)	Nein, das geht nicht. (VC7)<br>Nein, das geht nicht. (VC8)
	Yes	Ja, die kannst du für ein paar Tage haben, ich muss in der nächsten Zeit nichts bohren. (VC3)<br>Ja, die kannst du für ein paar Tage haben, die liegt bei mir eh nur rum. (VC4)	Nein, das geht nicht, weil ich keine habe. (VC1)<br>Nein, das geht nicht, weil ich die gerade selber brauche. (VC5)
Scenario 8		Für nächsten Samstagnachmittag plane ich ein Kaffeetrinken, um ein paar Nachbarn näher kennen zu lernen. Magst du dazu kommen?	
Explanation	No	Ja, ich komme dazu. (VC2)<br>Ja, ich komme dazu. (VC3)	Nein, da komme ich nicht. (VC5)<br>Nein, da komme ich nicht. (VC6)
	Yes	Ja, ich komme dazu, da habe ich noch nichts vor. (VC1)<br>Ja, ich komme dazu, da habe ich gerade frei bekommen. (VC7)	Nein, da komme ich nicht, ich muss Samstagnachmittags immer arbeiten. (VC4)<br>Nein, da komme ich nicht, da bin ich schon verabredet. (VC8)
Scenario 9		Vielen Dank für deine Antworten, wollen wir uns hinsetzen und noch etwas plaudern?	
Explanation	No	Ja, das können wir machen. (VC2)<br>Ja, das können wir machen. (VC5)	Nein, das geht nicht. (VC1)<br>Nein, das geht nicht. (VC6)
	Yes	Ja das können wir machen, ich habe eh noch Zeit zu überbrücken, bis ich verabredet bin. (VC3)<br>Ja das können wir machen, ich habe gerade eh nichts anderes zu tun. (VC7)	Nein, das geht nicht, ich bin müde und muss morgens immer früh raus. (VC4)<br>Nein, das geht nicht, ich muss nämlich direkt weiter. (VC8)

Note: VC: virtual character

Table S3

Answer scheme

Scenario / Character	1	2	3	4	5	6	7	8	9
1	an	ry	ay	an	rn	rn	ry	ay	rn
2	rn	ry	ry	ay	an	ry	an	an	an
3	ay	rn	rn	an	ry	ry	ay	an	ay
4	ry	an	rn	ry	an	an	ay	ry	ry
5	an	ay	ay	ry	rn	an	ry	rn	an
6	rn	ay	ry	rn	ay	ay	an	rn	rn
7	ay	rn	an	ay	ry	rn	rn	ay	ay
8	ry	an	an	rn	ay	ay	rn	ry	ry

Note. an = acceptance without explanation; ay = acceptance with explanation, rn = rejection without explanation, ry = rejection with explanation.

Table S4

Sample characteristics of analyzed sample

	HC (N = 32)	BPD (N = 30)	test statistics	p-value	α (HC; BPD)
age	28.19 (8.14)	29.93 (13.33)	$t = .62$	.540	
sex assigned by birth	84.4% female	86.7% female	$\chi^2 = 0$	1.000	
relationship	53.13% single	60.0% single	$\chi^2 = .08$	.772	
educational level [a]			Fisher's exact test, two-sided	.440	
low	0 %	10%			
intermediate	31.25 %	30%			
high	68.75 %	60%			
occupation			Fisher's exact test, two-sided	< .001	
unemployed	3.13%	33.3%			
student/pupil	53.13%	36.7%			
(self-) employed	43.75%	30%			
current treatment	0%	90%	Fisher's exact test, two-sided	< .001	
PAI-BOR	19.59 (6.71)	52.47 (7.96)	$t = 17.52$, $d = 4.48$	< .001	.94 (.68; .75)
BSL23	0.17 (0.16)	2.01 (0.97)	$t = 10.11$, $d = 2.69$	< .001	.98 (.75; .96)
BDI II	4.69 (4.12)	33.53 (11.35)	$t = 13.13$, $d = 3.42$	< .001	.97 (.81; .90)
Loneliness[b]	8.55 (2.44)	10.66 (2.45)	$t = 6.29$, $d = 0.86$	< .001	.91 (.86; .84)
Self-esteem	25.09 (6.68)	10.23 (4.80)	$t = -13.62$, $d = -2.54$	< .001	.95 (.80; .82)
Rejection sensitivity[b]	6.21 (3.05)	15.66 (8.55)	$t = 5.63$, $d = 1.49$	< .001	.93 (.83; .89)
Internal Attribution negative[b]	64.13 (10.41)	81.52 (15.18)	$t = 5.17$, $d = 1.34$	< .001	.88 (.79; .84)
Internal Attribution positive[b]	75.75 (8.60)	55.28 (14.57)	$t = -6.60$, $d = -1.73$	< .001	.88 (.72; .82)
ACE severity[b]	32.22 (9.34)	59.86 (19.18)	$t = 7.04$, $d = 1.85$	< .001	.96 (.91; .93)

Note. a = refers to the German school qualification system, low = less than 10 years of school education, intermediate = 10 years of school education, high = 12 – 13 years of school education. b = missing data of one BPD participant. PAI-BOR = Personality Assessment Inventory Borderline Scale, BSL23 = Borderline Symptom List, BDI II = Beck Depression Inventory.

Table S5

Descriptive statistics of benevolence ratings

	HC (n = 32)		BPD (n = 30)	
	Mean	*SD*	Mean	*SD*
rejection: unexplained	8.91	2.19	7.89	2.63
rejection: explained	11.17	1.64	9.07	2.07
acceptance: unexplained	12.98	2.31	10.40	1.79
acceptance: explained	12.58	2.44	9.78	1.87

Note. *SD* = standard deviation.

Repetition of mixed-effects model analyses with data of the complete sample

Model specification was as follows: rating ~ group * reaction * explanation + (1 | subject) + (1|subject:reaction) + (1|subject:reaction:explanation) + (1|character) + (1|question). The model's total explanatory power is substantial (conditional R^2 = 0.541). The proportion of explained variance related to the fixed effects alone (marginal R^2) was 17.7%, indicating that 36.4% of the variance in benevolence ratings was explained by the CRIs. Model comparison revealed a significant better model fit compared to the null model without any fixed effects ($\chi^2(7) = 119.66$, $p < .001$). The model's intercept, corresponding to the grand mean, is at 10.37. Benevolence ratings were 2.10 scores lower for participants of the BPD group (main effect of group). Both acceptance and providing an explanation (main effects of reaction and explanation) increased benevolence ratings. However, in situations of acceptance providing an explanation did not result in higher or lower benevolence ratings (interaction reaction × explanation). Post hoc analyses of the model's marginal means revealed that benevolence ratings after rejection trials were higher if an explanation was given (Z = -7.76, $p_{FDR} < .001$), while providing an external explanation did not influence benevolence ratings in acceptance trials (Z = 0.42, p_{FDR} = .676). The marginal significant interaction effect of group × explanation indicated that the effect of explanation differed between the groups. Post hoc analyses of the model's marginal means revealed that providing an external explanation increased benevolence ratings in BPD patients less strong than in HC (HC: Z = -5.05, $p_{FDR} < .001$; BPD: Z = -2.41, p_{FDR} = .016). For further details see Table S3.

Table S6

Fixed effects for linear mixed model predicting benevolence ratings

	Estimate	95% CI	Std. Error	*df*	*t*	*p*
Intercept	10.37	9.46, 11.27	0.46	17.87	22.36	< .001
Group	1.05	0.62, 1.47	0.22	62.01	4.84	< .001
Reaction	-1.25	-1.56, -0.94	0.16	62.13	-7.90	< .001
Explanation	-0.38	-0.52, -0.24	0.07	124.37	-5.27	< .001
Reaction × Explanation	-0.42	-0.57, -0.28	0.07	139.22	-5.71	< .001
Group × Reaction	-0.12	-0.44, 0.19	0.16	62.02	-0.79	.435
Group × Explanation	-0.13	-0.28, 0.01	0.07	123.37	-1.87	.064
Group × Reaction × Explanation	-0.08	-0.22, 0.06	0.07	123.37	-1.15	.251

Note. Estimates represent effect-coded parameters, therefore the higher factor level (group = BPD, Reaction = acceptance, explanation = yes) receives a value of -1 and the lower level (group = HC, reaction = rejection, explanation = no) a value of 1. Main effects correspond to half of the difference between the two conditions.

Results of cumulative link mixed-model

Prediction of level of benevolence rating by group, reaction and explanation

We fitted a cumulative link mixed model with Laplace approximation to predict benevolence ratings with group, reaction and explanation as well as their interaction terms. Since the ordinal package can not work with complex random intercepts, we rebuild our model with random slopes. The model specification was as follows: rating ~ group * reaction * explanation + (1 + reaction + reaction:explanation | subject) + (1 | character) + (1 | question). The model's total explanatory power is substantial (conditional R^2 = 0.528) and the proportion of explained variance related to the fixed effects alone (marginal R^2) was 18.7%, indicating that 34.1% of the variance in benevolence ratings was explained by the random effects structure. The results replicated our findings from the continuous model, fixed-effects can be found in Table S4. The threshold coefficients, representing the boundary estimates for each of the ordinal levels of the dependent variable as adjusted log-odds, can be found in Table S5. Model comparison revealed a significant better model fit compared to the null model without any fixed effects ($\chi^2(7) = 79.48$, $p < .001$).

Table S7

Fixed effects for cumulative link mixed model predicting benevolence ratings

	Estimate	Std. Error	*z*	*p*
Group	0.74	0.15	4.83	< .001
Reaction	-0.74	0.08	-9.00	< .001
Explanation	-0.23	0.05	-4.97	< .001
Reaction × Explanation	-0.27	0.05	-5.24	< .001
Group × Reaction	-0.21	0.08	-2.53	.011
Group × Explanation	-0.12	0.05	-2.65	.008
Group × Reaction × Explanation	-0.06	0.05	-1.22	.221

Note. Estimates represent effect-coded parameters.

Table S8

Threshold coefficients of cumulative link mixed model predicting benevolence ratings

	Estimate	Std. Error	*z*
0|1	-6.39	0.35	-18.16
1|2	-5.52	0.33	-16.68
2|3	-4.79	0.32	-14.92
3|4	-4.15	0.32	-13.17
4|5	-3.61	0.31	-11.59
5|6	-3.09	0.31	-9.97
6|7	-2.55	0.31	-8.27
7|8	-1.96	0.31	-6.38
8|9	-1.33	0.31	-4.35
9|10	-0.62	0.31	-2.03
10|11	0.15	0.31	0.49
11|12	0.84	0.31	2.75
12|13	1.52	0.31	4.97
13|14	2.15	0.31	6.99
14|15	2.76	0.31	8.95
15|16	3.40	0.31	10.92
16|17	4.08	0.31	12.98
17|18	4.69	0.32	14.68
18|19	5.38	0.33	16.39
19|20	6.03	0.34	17.73

Table S9

Model comparison of null and full mixed model predicting benevolence ratings

	**null model**			**full model**		
Predictors	*Estimates*	*CI*	*p*	*Estimates*	*CI*	*p*
(Intercept)	10.38	9.42 – 11.35	**<0.001**	10.35	9.42 – 11.27	**<0.001**
Group				1.06	0.63 – 1.50	**<0.001**
Reaction				-1.10	-1.33 – -0.86	**<0.001**
Explanation				-0.35	-0.49 – -0.21	**<0.001**
Group × Reaction				-0.28	-0.51 – -0.05	**0.019**
Group × Explanation				-0.16	-0.30 – -0.02	**0.023**
Reaction × Explanation				-0.40	-0.55 – -0.25	**<0.001**
(Group × Reaction) × Explanation				-0.10	-0.24 – 0.04	0.151
**Random Effects**						
σ^2	7.72			7.72		
τ_{00}	1.44 Subject:Reaction:Explanation			0.85 Subject:Reaction:Explanation		
	3.38 Subject:Reaction			1.12 Subject:Reaction		
	2.00 Subject			2.17 Subject		
	0.34 Question			0.34 Question		
	1.11 Character			1.08 Character		
ICC	0.52			0.42		
N	62 Subject			62 Subject		
	2 Reaction			2 Reaction		
	2 Explanation			2 Explanation		
	8 Character			8 Charater		
	9 Question			9 Question		
Observations	4437			4437		
Marginal R^2 / Conditional R^2	0.000 / 0.517			0.172 / 0.519		

Table S10

Correlation coefficients between benevolence ratings and Attributional style, self-esteem, ACE, rejection sensitivity and loneliness

**Benevolence ratings**		Internality positive events	Internality negative events	Rejection sensitivity	Self-esteem	ACE severity	loneliness
across conditions	HC	.073	-.233	-.269	-.021	-.270	.016
	BPD	.260	-.184	-.174	.280	-.273	-.313[a]
rejection	HC	.106	-.018	-.061	-.132	-.106	-.035
	BPD	.199	-.171	-.209	.261	-.270	-.297
acceptance	HC	.031	-.292	-.313	.054	-.286	.042
	BPD	.319[a]	-.189	-.118	.283	-.285	-.311
without explanation	HC	.128	-.116	-.222	-.052	-.227	-.027
	BPD	.216	-.156	-.188	.256	-.273	-.320[a]
with explanation	HC	.018	-.298	-.272	.007	-.269	.048
	BPD	.304	-.212	.154	.300	-.267	-.298
acceptance minus rejection	HC	-.033	-.275	-.269	.131	-.217	.061
	BPD	.108	.044	.236	-.085	.134	.104
with minus without explanation	HC	-.121	-.292	-.132	.071	-.122	.099
	BPD	.195	-.115	.168	.037	.110	.178

Note. Estimates represent Pearson correlations, a = $p < .1$

Table S11

Correlation coefficients between Attributional style, self-esteem, ACE, rejection sensitivity and loneliness

		Internality positive events	Internality negative events	Rejection sensitivity	Self-esteem	ACE severity	loneliness
internality positive events	*r*		-.005	-.277	.292	-.152	-.235
	p		.977	.125	.105	.405	.195
internality negative events	*r*	**-.638**		.242	-.281	.000	-.045
	p	< .001		.182	.119	.999	.808
rejection sensitivity	*r*	-.159	.243		-.127	**.685**	**.563**
	p	.410	.203		.487	< .001	<.001
self-esteem	*r*	**.485**	**-.623**	**-.434**		.069	-.075
	p	.008	< .001	.019		.708	.683
ACE severity	*r*	-.240	.056	**.457**	-.246		**.571**
	p	.210	.774	.013	.198		< .001
loneliness	*r*	-.188	**.373**	**.377**	**-.497**	**.432**	
	p	.329	.046	.044	.006	.019	

Note. Above diagonal = HC, below diagonal = BPD.

Association of benevolence ratings with Attributional style

Multiple linear regression revealed that neither in the BPD group, nor in the HC group, neither the difference in benevolence ratings between acceptance and rejection, nor the difference in benevolence ratings between trials with and without explanation was predicted by attributing positive or negative events internally (difference between reaction: HC: $F(2, 29) = 1.20$, $R^2 = .07$, $p = .315$; BPD: $F(2, 26) = 0.45$, $R^2 = .03$, $p = .644$; difference between explanation: HC: $F(2, 29) = 1.62$, $R^2 = .10$, $p = .216$; BPD: $F(2, 26) = 0.52$, $R^2 = .04$, $p = .603$). For further details see tables S12 and S13.

Table S12

Prediction of difference in benevolence ratings between acceptance and rejection trials by attributing positive and negative events internal in the HC and BPD subsamples

	HC			*BPD*		
Predictor variables	*β*	*t*	*p*	*β*	*t*	*p*
internal Attribution positive events	-0.08	-0.19	.849	0.26	0.92	.368
internal Attribution negative events	-0.65	-1.54	.134	0.21	0.76	.453

Note. Predictors were standardized.

Table S13

Prediction of difference in benevolence ratings between trials with and without an external explanation by attributing positive and negative events internal in the HC and BPD subsamples

	HC			*BPD*		
predictor variables	*β*	*t*	*p*	*β*	*t*	*p*
internal Attribution positive events	-0.15	-0.70	.491	0.13	0.82	.420
internal Attribution negative events	-0.36	-1.66	.108	0.01	0.06	.951

Note. Predictors were standardized.

Association of benevolence ratings with severity of ACE, rejection sensitivity, self-esteem and loneliness

The severity of ACE, rejection sensitivity and self-esteem did not predict the difference in benevolence ratings between acceptance and rejection trials, neither in the HC, nor in the BPD group (HC: $F(3, 28) = 0.88$, $R^2 = .09$, $p = .462$; BPD: $F(3, 25) = 0.50$, $R^2 = .06$, $p = .687$). Similarly, the severity of ACE, rejection sensitivity and self-esteem did not predict the difference in benevolence ratings between trials with and without an external explanation, neither in the HC, nor in the BPD group (HC: $F(3, 28) = 0.22$, $R^2 = .02$, $p = .879$; BPD: $F(3, 25) = 0.37$, $R^2 = .04$, $p = .776$). For further details see tables S14 and S15.

Table S14

Prediction of difference in benevolence ratings between acceptance and rejection trials by self-esteem, rejection sensitivity and childhood trauma in the HC and BPD subsamples

	HC			*BPD*		
	β	*t*	*p*	*β*	*t*	*p*
self-esteem	0.27	0.61	.549	0.00	-0.02	.988
rejection sensitivity	-0.45	-0.74	.463	0.25	0.93	.361
ACE severity	-0.22	-0.37	.712	0.04	0.15	.883

Note. Predictors were standardized.

Table S15

Prediction of difference in benevolence ratings between rejection trials with and without an explanation by self-esteem, rejection sensitivity and childhood trauma in the HC and BPD subsamples

	HC			*BPD*		
	β	*t*	*p*	*β*	*t*	*p*
self-esteem	0.08	0.35	.730	0.08	0.58	.567
rejection sensitivity	-0.09	-0.26	.797	0.12	0.84	.407
ACE severity	-0.10	-0.30	.765	0.03	0.22	.824

Note. Predictors were standardized.

Multiple linear regression revealed that the difference in benevolence ratings between acceptance and rejection trials as well as the difference in benevolence ratings between trials with and without an external explanation did not predict the level of loneliness neither in the HC, nor in the BPD Group (HC: $F(2, 29) = 0.17$, $R^2 = .01$, $p = .843$; BPD: $F(2, 26) = 0.44$, $R^2 = .03$, $p = .650$). When adding the severity of ACE, rejection sensitivity and self-esteem as additional predictors, the model explained 44.44% of the variance in loneliness in the HC group ($F(5, 26) = 4.16$, $p = .007$, adjusted $R^2 = .34$). However, higher ACE severity was the only marginally significant predictor for higher loneliness in the HC group ($\beta = 0.24$, $p = .068$). Please note that in the HC group, rejection sensitivity and severity of ACE were highly correlated ($r = .685$, $p < .001$). In the BPD group, the severity of ACE, rejection sensitivity, self-esteem and difference in benevolence ratings between acceptance and rejection trials as well as the difference in benevolence ratings between trials with and without an external

explanation explained 39.26% of the variance in loneliness ($F(5, 23) = 2.97$, $p = .033$, adjusted $R^2 = .26$). Lower self-esteem was the only significant predictor for higher loneliness in BPD ($\beta = -0.27$, $p = .028$). For further details see table S15.

Table S16

Prediction of loneliness by self-esteem, rejection sensitivity, childhood trauma and differences in benevolence ratings between rejection and acceptance trials and trials with and without an explanation in the HC and BPD subsamples

	HC			*BPD*		
	β	*t*	*p*	*β*	*t*	*p*
self-esteem	-0.06	-0.61	.550	**-0.27**	**-2.37**	**.028**
rejection sensitivity	0.22	1.69	.103	0.03	0.26	.795
ACE severity	0.24	1.90	.068	0.19	1.62	.118
acceptance - rejection	0.18	1.03	.313	-0.13	-0.85	.404
with – without explanation	-0.03	-0.18	.860	0.19	1.24	.227

Note. Predictors were standardized.
